# Influence of controlled environmental conditions in potential salivary ocular pain biomarkers for enhancing the assessment of ocular pain

**DOI:** 10.1371/journal.pone.0296764

**Published:** 2024-01-26

**Authors:** Eva M. Sobas, Vázquez Amanda, Itziar Fernández, Roberto Reinoso, Carmen García-Vázquez, Enrique Ortega, Amalia Enríquez-de-Salamanca

**Affiliations:** 1 Institute of Applied Ophthalmobiology (IOBA), University of Valladolid, Valladolid, Spain; 2 OculoFacial Pain Unit, IOBA, University of Valladolid, Valladolid, Spain; 3 Nursery Faculty, University of Valladolid, Valladolid, Spain; 4 Networking Research Center on Bioengineering, Biomaterials and Nanomedicine (CIBER-BBN), Carlos III National Institute of Health, Valladolid, Spain; 5 Deparment of Didactics of Experimental Sciences, Social Sciences and Mathematics, Faculty of Education and Social Work, University of Valladolid, Valladolid, Spain; 6 Pain Unit, Aliance of University Hospitals, Castile and Leon Nacional Health System, Valladolid, Spain; University of Pisa, ITALY

## Abstract

**Purpose:**

We endeavored to identify objective salivary biomarkers for pain, a subjective sensation with a biological basis, using molecules already described related to pain. The study aimed to analyze inter-individual differences and intersession variability in salivary potential ocular pain biomarkers on healthy subjects, in samples obtained under the influence of controlled environmental conditions.

**Methods:**

Thirty-four healthy subjects, 20 male, 14 female, median age 35.44 years (range 30–40) were exposed for 30 minutes under standard environmental conditions (T: 22°C, 50% relative humidity) in the Controlled Environmental Research Laboratory (CE-Lab, Vision R&D, Valladolid Spain) in two separate visits (V1, V2) at least 24 hours apart. Saliva was collected after the exposure in each of the visits, and cortisol, α-amylase (sAA), secretory IgA (sIgA), testosterone, and soluble fraction of TNFα receptor II (sTNFαRII) were analyzed by ELISA. Repeatability of inter-subject inter-session measurements was assayed by intraclass correlation coefficient (ICC).

**Results:**

There were no significant inter-session differences in testosterone (p = 0.2497), sTNFαRII (p = 0.6451) and sIgA (p = 0.9689) salivary levels. The reproducibility for salivary cortisol, sAA, testosterone, sTNFαRII and sIgA were 0.98 ng/ml, 20.58 U/ml, 21.07 μg/ml, 24.68 pg/ml and 0.19 pg/ml, respectively. Salivary cortisol, sAA, testosterone, sTNFαRII and sIgA yielded the following ICCs: 0.506, 0.569, 0.824, 0.870 and 0.4295, respectively; all these ICCs (except that for cortisol and sIgA) were found to be improved compared to those found previously by our group in a previous study in salivary samples obtained from healthy subjects under non-controlled environmental conditions; Cortisol´s ICC didn´t improve and was in both cases at the limit of acceptability.

**Conclusion:**

Environmental factors such as temperature and relative humidity affect the reproducibility of measurement of some salivary molecules which have been proposed as potential pain biomarkers. The exposure of subjects to standard controlled environmental conditions before salivary sample obtention would improve the reproducibility of these molecule measures’ as potential biomarkers of chronic ocular pain.

## Introduction

Chronic pain affects hundreds of millions of people worldwide [1]. In the evaluation of pain, various tools are available for use, including self-reported pain assessments, direct observation of an individual’s behavior, and the examination of biomarkers. Over the last few decades, there has been a growing interest in utilizing saliva as a means to identify and quantify biomarkers associated with pain [2]. The salivary glands are integrated into the neuroendocrine system and contain a wide array of molecules that might play important roles in the pathophysiology of chronic pain conditions [3].

A biomarker is defined as a marked characteristic that is measured as an indicator of normal biological processes, pathogenic processes or responses to an exposure or intervention [4]. Effective biomarkers ought to exhibit minimal intersubject or interday variability, possess a significant signal-to-noise ratio, and demonstrate prompt and consistent changes in response to alterations in the condition or its treatment [5]. Saliva is ideal for early detection of disease as it contains specific soluble biomarkers [6]. Saliva is easily collected in a noninvasive and not painful manner [3]. Therefore, the determination of biomarkers of pain in saliva would be an enormously useful, noninvasive and economical tool [7].

Climatological effects can affect human parotid gland function. It has been described that the human parotid gland can be sensitive to dehydration [8]. The parotid fluid flow rate seems to be higher in spring and tends to decrease in late autumn or winter [8]. Thus, the climatological conditions such as the heat in summer should be a factor to consider as it can influence the levels of secretion and gland function.

Hence, investigating salivary biomarkers while controlling environmental factors would mitigate the impact of unfavorable conditions, both external (weather-related conditions) and internal (controlled environmental conditions). Achieving standardized environmental conditions, including temperature, humidity, airflow, and possibly barometric pressure, is attainable by utilizing Controlled Environment Laboratories, where continuous regulation is implemented [9, 10].

The purpose of this study was to analyze inter-individual differences and inter-session variability in several molecules which have been proposed as potential ocular pain biomarkers in saliva on healthy subjects under controlled environmental conditions of temperature and relative humidity; the results obtained were compared with those from a previous study of our group in which these values were analyzed under non-controlled environmental conditions.

## Patients and methods

### Ethics statement

This prospective study followed the principles outlined in the Declaration of Helsinki, with informed consent being acquired from the participants after providing them with a thorough explanation of the study’s purpose and potential outcomes. Furthermore, the study received approval from the Ethics Committee at the University of Valladolid.

### Participants and study design

This was a prospective, descriptive study. Healthy subjects between 30 and 40 years old were recruited. Exclusion criteria for all participants were pain of any origin, diagnosed autoimmune disease, treatment with corticosteroids, non-steroidal anti-inflammatory drugs or analgesics, smokers, pregnancy, breastfeeding, or women under hormonal treatment. Oral diseases with inflammation or active lesions of the mouth were also exclusion criteria [11].

Participants were evaluated at Controlled Environmental Research Laboratory (CE-Lab, Vision R&D, Valladolid Spain) and they remained for 30 min there under standard environmental conditions (T° = 22°C, 50% relative humidity) prior to the salivary sample collection.

Two samples of saliva were collected from each subject in two separate visits (V1, V2). Samples collected in V2 were always obtained 23–25 hours after the ones collected in the first visit.

The samples data used in this study can be found in the following repository [12]. S1 Table. Saliva sample results in healthy subjects in the Controlled Environmental Research Laboratory.

### Sample collection

Saliva samples were obtained using the passive secretion method over 5 minutes. The procedure employed for sample collection has been previously detailed in prior academic publications [11, 13]. Previously to the visit, participants were instructed how to collect their saliva samples. Additionally, the professionals were also instructed on how to perform the procedure. The minimum allowed amount of saliva to be collected was 1 ml. If the subject filled the 5 ml before 5 minutes, the time was noted to calculate the flow rate. The sample was discarded if it was contaminated with blood. In that case, a new collection was repeated after 10 minutes. After collection, the samples were frozen at -20°C until analysis.

All saliva samples collections were conducted, under supervision of one of the co-authors, between 10:00 a.m and 12:00 a.m to minimize the effect of the hormonal diurnal circadian rhythm.

The data of the menstrual cycle in women was collected to review the differences due to hormonal fluctuations. The time collection, time since the last meal, volume and the flow rate were included.

### Determination of salivary molecules

Cortisol, α-amylase (sAA), secretory IgA (sIgA), testosterone, and soluble fraction of TNFα receptor II (sTNFαRII) were analyzed in salivary samples by enzyme-linked immunosorbent assay (ELISA) using the commercially available kits: Cortisol (DRG® Salivary Cortisol ELISA, DRG® Instruments GmbH, Marburg, Germany), testosterone (DRG® Salivary Testosterone ELISA, DRG Instruments GmbH), sAA (DRG Salivary Alpha Amylase ELISA, DRG Instruments GmbH), sTNFαRII (Quantikine®, Human sTNF RII/TNFRSF1B Immunoassay, R&D Systems, Minneapolis, MN, USA) and sIgA (Salimetrics® Salivary Secretory IgA ELISA, Pennsylvania, USA). The samples were analyzed following manufacturer’s instructions. The SpectraMAX® M5 multidetection microplate reader and the SoftMax Pro 4.8 software used to analyze ELISA results were from Molecular Devices (Sunnyvale, CA). The minimum detectable doses for the human Cortisol, α-amylase (sAA), secretory IgA (sIgA), testosterone, and soluble fraction of TNFα receptor II (sTNFαRII) ELISAs were 0.09 ng/ml, 1U/ml, 2.5 μg/ml, 1.9 pg/mL, and 1.0 pg/mL, respectively, according to each ELISA kit’s instructions.

### Data analysis

Statistical analysis was carried out using R (version 3.6.1) [14], and Package irrR [15] was used for intraclass correlation coefficient (ICC) estimations. For all cases, a significance of 0.05% was assumed.

Quantitative variables were presented as means with standard deviations (SDs) or medians with ranges, determined by the distribution’s normality, while qualitative variables were expressed as percentages. In each instance, 95% confidence intervals (CIs) were established.

In addition, for each of the variables collected in the two measurements, a comparison of the measurements was made using the t-Student test for the quantitative variables whenever it is possible to assume normality in the differences. In other cases, the Wilcoxon test was used.

To assess agreement of measurements between salivary collections at both visits we used Bland-Altman plots and limits of agreement (LoA). LoAs were characterized as the average variation in measurements conducted during two distinct sessions, within a range of ±2 times the SD. Here, SD represents the observed standard deviation of the discrepancies between the two measurements for each subject.

To evaluate the reproducibility of each salivary biomarker, the within-subject standard deviation (Sw) was calculated by obtaining the square root of the sum of the within-subject variance and the error variance estimated in a linear random-effects model [16]. That is, the spread of the measurements from different saliva collections on the same subject. The precision (1.96 × Sw) and the reproducibility (2.77 × Sw) were calculated as previously reported (13–15). In addition, the within-subject coefficient of variation (CVw) was calculated. CVws were defined on the original scale, using the expression 10Sw—1, where Sw is calculated using log transformed data [17, 18].

Repeatability of inter-subject inter-day measurements was assayed by intraclass correlation coefficient (ICC). To obtain ICC estimator with its confidence interval (CI), the Package irrR [15] is used. The interpretation was made under Portney classification: 0–0.2, poor agreement; 0.3–0.4, fair agreement; 0.5–0.6, moderate agreement; 0.7–0.8, strong agreement; and >0.8, almost perfect agreement).

## Results

### Sample demographic characteristics

Initially, forty-one healthy subjects were recruited. Out of those 41, four subjects were discarded at the V2, two due to be not compatible with the timetable and two due to pain and having ingested analgesics in the 24 hours prior to the sample. Three other subjects were as well excluded of the study because their saliva samples were not processable. For these reasons, the final sample size was reduced to 34 subjects.

Fourteen participants in the study were men and twenty women. The median age was 35.4 years old (range 30–40).

Out of the twenty women, seven were in follicular phase, eight in luteal phase, and none was in the hemorrhagic phase; there were five women for whom the information about menstrual cycle was not available on any of the visits.

### Saliva collection characteristics

The median collection time was 300 sec (range 138–300 sec) for the first collection (in V1) and 300 sec (range 240–300 sec) for the second (in V2) (p = 0.2473). The median collection volume was 2.5 mL (range 0.5–4.5 mL) for the first collection and 3.0 mL (range 0.9–4.5 mL) for the second (p = 0.0265). The median salivary flow rate was 0.50 mL/min (range 0.10–1.15 mL/min) for the first collection and 0.60 mL/min (range 0.18–1.16 mL/min) for the second (p = 0.3474).

The median elapsed time since the last meal before collection was 146min (range 60–780 min) for the first saliva collection in V1 and 147 min (range 92–783 min) for the second one in V2 (p = 0.2381).

### Salivary molecule analysis

Cortisol concentration was significantly higher at V1 compared to V2, whereas the sAA concentration was lower at the V1 compared to V2. There were no significant differences between V1 and V2 levels of sIgA, total testosterone and sTNFαRII salivary concentration. Although, testosterone concentration was found significantly higher in females in V1 compared to V2 Table 1.

**Table 1:** Differences of salivary molecules concentrations between collections.

Molecule	1^st^ Salivary sample collection (S1)		2^nd^ Salivary sample collection (S2)		Mean of difference S2-S1	95% CI for the mean of difference	p-value
	Concentration	95%CI	Concentration	95%CI			
Cortisol (ng/ml)	7.79 ± 2.07	7.07–8.51	7.11 ± 1.71	6.52–7.71	-0.68	-1.31, -0.05	**0.0358**
sIgA (μg/mL)	181.04 ± 151.09	128.33–233.76	190.34 ± 192.58	123.15–257.53	9.3	53.73, 72.33	0.0689
sAA *U/mL*	42.76 ± 32.18	31.54–53.99	61.15 ± 42.72	46.25–76.06	+18.39	+3.09, +33.70	**0.0002**
Testosterone *(males*, *n = 14)* ng/mL	152.39 ± 47.82	124.78–180	155.49 ± 57.93	122.04–188.94	+3.10	-25.33, +31.53	0.8174
Testosterone *(females*, *n = 20)* ng/mL	84.03 ± 41.78	64.47–103.58	69.01 ± 31.69	54.18–83.84	-15.01	-26.97, -3.05	**0.0166**
Testosterone *(total)* ng/mL	112.18 ± 55.43	92.84–131.52	104.62 ± 61.37	83.21–126.03	-7.55	-20.67, +5.56	0.2497
sTNFαRII pg/mL	106.28 ± 139.36	57.65–154.9	105.20 ± 136.43	57.60–152.80	-1.08	-18.77, +16.61	0.6451

n = 34 for each salivary molecule; CI, confidence interval; values for all salivary molecules were calculated regardless of sex of the subject, except for testosterone.

**Fig 1:**
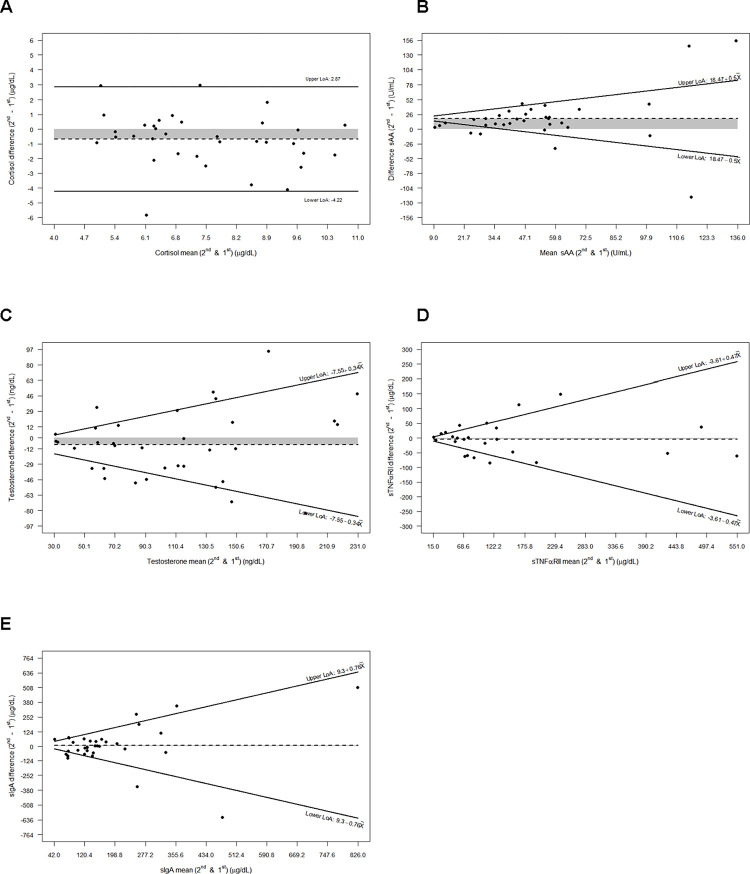
Difference versus mean for each in molecule between visits. Bland-Altman graphs showing the intersession reproducibility for each biomarker: (A) Cortisol (B) sAA, (C) Testosterone, (D) sIgA, and (E) sTNFαRII. The solid lines represent the upper and the lower LoA (limits of agreement): crude 95%. LoA are depicted in A and the 95% back transformed LoA after the 10 base log-transformation are shown as functions of the mean of the two measurements in the remaining (X denotes the corresponding biomarker mean). Dashed line represents the mean difference value between 2nd and 1^st^ salivary collections, and shaded area the magnitude between this mean difference value and zero.

To evaluate the difference versus mean for each molecule between visits, the Bland-Altman plots was calculated. Fig 1 shows that the difference in the mean for molecules measures between V1 and V2 was significant for cortisol (p = 0.0358) and sAA (p = 0.0002). However, testosterone (p = 0.0753), sTNFαRII (p = 0.7009) and sIgA (p = 0.9689), showed no significant differences.

The intersession within-subject standard deviation (S_w_), the precision, the reproducibility, and the within-subject coefficient of variation (CV_w_), of each molecule were calculated Table 2.

**Table 2:** Intersession within-subject standard deviation, precision, reproducibility and within-subject coefficient of variation for the intersession analysis of each biomarker.

Molecule	S_w_ (95%CI)	Precision S_w_*1.96	Reproducibility S_w_*2.77	CV_w_ (%) (95% CI)
Cortisol	0.98 (0.65;1.31)	1.92	2.71	13.47 (8.63;18.31)
sAA	20.58 (11.36;28.81)	40.34	57.01	37.83 (27.83;47.80)
Testosterone	21.07 (15.25;26.90)	41.3	58. 36	20.06 (15.31;24.81)
sTNFαRII	24.68 (15.73;33.63)	48.37	68.36	46.48 (30.20;62.76)
sIgA	0.19 (0.13; 0.25)	0.37	0.53	9.12 (6.05; 12.19)

S_w_: within-subject standard deviation; CV_W_: within-subject coefficient of variation; sAA: α-amylase; sIgA: secretory IgA; sTNFαRII: soluble fraction of receptor II of tumor necrosis factor α.

To analyze the intersession reproducibility the ICC was calculated for each molecule. sTNFαRII and testosterone had the highest values, 0.870 and 0.824 respectively, indicating very good intersession reproducibility. sAA and cortisol ICC values 0.569 and 0.506 respectively), indicated moderate reproducibility at the limit of acceptability. sIgA had the lowest value, 0.4295, which indicated fair reproducibility. Table 3 shows these results.

**Table 3:** Reliability of molecules to be considered potential ocular pain biomarkers.

Molecule	ICC	95% CI ICC	Reproducibility Rating
Cortisol	0.506	0.210–0.717	Moderate
sAA	0.569	0.289–0.761	Moderate
Testosterone	0.824	0.678–0.908	Very good
sTNFαRII	0.870	0.730–0.940	Very good
sIgA	0.429	0.1151–0.6666	fair

n = 34 for each biomarker; ICC: intraclass correlation coefficient; CI: confidence interval; sAA: α-amylase; sIgA: secretory IgA; sTNFαRII: soluble fraction of receptor II of tumor necrosis factor α.

## Discussion

The present study has studied the reliability and reproducibility of the measurement in two separate visits of several salivary molecules (i.e. cortisol, sAA, sIgA, testosterone and sTNFaRII, which have been proposed as potential pain biomarkers in saliva samples) under standard controlled environmental conditions. Under these conditions ICC values for Testosterone, sTNFaRII were found to have a very good reproducibility, whereas those of cortisol and sAA were just moderate, and fair for sIgA. However, sIgA showed the best within-subject standard deviation and the best within-subject coefficient of variation.

Testosterone, sAA, and sTNFaRII presented ICC values higher (i.e. better) than those found previously by our group, determined in the samples from healthy subjects under non-controlled environmental conditions [11]. Particularly sAA and testosterone reliability improved considerably, particularly sAA ICC from “very poor” to “moderate”, and testosterone from “fair” to “very good”. Cortisol remained moderate and sIgA was the only one that decreases reliability. This indicates that controlling environmental conditions while taking the saliva samples improves the reliability of potential biomarker’s measurement.

Some authors consider sAA to be a sensitive biomarker of stress-related changes that reflects the activity of the sympathetic nervous system (SNS) [19–21]. Furthermore, chronic pain is associated with psychological problems, specifically personality and mood changes, anxiety and depression [22]. Other authors conclude that there is insufficient support for the use and interpretation of sAA activity as a valid and reliable measure of SNS activity [23]. In our previous study, when measured in saliva samples in subjects under non-controlled environmental conditions, sAA had the lowest reproducibility value among all of the rest of molecules analyzed [11]. But, as shown now by our results, this value increases when measured under standard controlled environmental conditions, in agreement with those previous reports that considered sAA to be an emerging biomarker for stress and pain, providing that it is analyzed in subjects under standard controlled environmental conditions.

Another molecule that has been proposed as potential pain biomarker is testosterone. Previous research has proposed that testosterone might play a part in reducing pain [24–26]. Even during the menstrual phase, it seems that elevated levels of natural testosterone in females play a pain-relieving function [26]. For this reason, this molecule has been considered of interest as a potential ocular pain biomarker, and we included it in our studies. The testosterone analysis in saliva has been validated in previous studies [24–27]. While serum is considered the optimal approach for hormonal assay due to its ability to capture blood-level fluctuations in testosterone more comprehensively, there have been reports indicating that saliva might not be as accurate in reflecting these changes [23]. Nevertheless, the author opted to utilize saliva samples in their research due to the benefit of circumventing the potential stress associated with the blood-draw technique [25].

Despite the bad result for testosterone ICC in our previous study in non-controlled environmental conditions, our new findings suggest that when measured in saliva samples obtained under standard controlled environmental conditions testosterone could be considered a good potential biomarker of ocular pain, as under these conditions testosterone ICC increases, and the variability of its levels in healthy, pain free subjects decreases.

Cortisol is another molecule whose measurement has been proposed as stress and pain biomarker. We found that it had acceptable levels of reproducibility regardless of environmental conditions, as shown in the present study, and also in our previous one [11]. However, cortisol levels showed more variability intersession under standard controlled environmental conditions than under a non-controlled condition. Previous studies by other authors have assessed the effect of cold environment exposure and cold acclimatization on its response [28]. Particularly, Izawa found that being exposed to cold temperatures raised cortisol levels in saliva [28]. However, this effect was not observed in individuals who had become accustomed to cold conditions. These findings align with previous studies that demonstrated a smaller cortisol response in individuals who were acclimated to the given conditions, while a greater response occurred in those who were not acclimated [29, 30]. In line with these conclusions, our results demonstrated that the subjects under standard control environmental conditions exhibited higher cortisol values than under non-controlled conditions. Taken together, these results could indicate that lower temperature and higher humidity could be the responsible of this variation in concentration.

Several studies have also pointed out the measurement of sTNFaRII as a pain biomarker [7, 31]. Our results, have shown that sTNFαRII behaves as a good potential biomarker regardless of environmental conditions in which it is collected [31, 32]. Moreover, its ICC slightly improves when measured in samples obtained under standard environmental controlled conditions.

Surprisingly, sIgA ICC values were better in samples collected in non-controlled environmental conditions in our previous study than under controlled conditions in our present study. The autonomic nervous system regulates the flow rate of saliva and the secretion of different salivary compounds [33]. Stimulation of sympathetic and parasympathetic neurons induces changes in saliva flow and the secretion of sIgA [34]. Since pain has the ability to impact sympathetic and parasympathetic activity, it serves as a stimulus capable of modifying salivary secretions [35].

Salivary flow can influence these molecules [35, 36]. On other hand, there are several factors that influence salivary flow rate: the heat in summer, the cold in winter, and to smoke [37]. With the use of controlled environmental conditions in the CAC and excluding smokers from the study we have managed these potential sources of measurement error with no significant difference in salivary flow rates between visits.

There are several studies on dry eye disease in which biomarkers were analyzed in tear samples of patients and healthy subjects under a controlled environment, either “normal” /standard (T: 22°C, 50% relative humidity) or “adverse” (T: range: 15–30°C, 1°C steps and relative humidity: range: 5%–80%, 1% steps) [9, 38, 39]. This demonstrates that environmental factors have been established as having an impact on the lacrimal functional unit [10, 38]. In a similar vein, environmental factors can also influence saliva. For example, Shannon et al. reported an association between heat exposure and a reduction in salivary flow rate [8]. Another study demonstrates that heat increases the secretion of IgA [40] and several authors demonstrated as well the influence of cold exposure on saliva cortisol values [28–30]. Consequently, the use of controlled environments during sample obtention would be recommended when potential biomarkers, at least those that we are measuring here, are analyzed.

The ultimate assessment of a biomarker is its ability to accurately forecast the desired result under real-life circumstances. Ideally, this is evaluated through meticulously planned randomized controlled clinical trials, and there are instances where such trials have demonstrated that a suggested biomarker is, in reality, invalid [5]. In certain cases, a way to avoid this problem could be the use of environmental controlled chambers. Environmental chambers recreate controlled environments to evaluate subjects in always the exact same conditions. This involves homogenization for sample collection, more similar response to predetermined stimuli and more consistent response to therapies [9]. Utilizing a controlled setting is advised for assessing the impacts of DED treatments and exploring the fundamental mechanisms of this condition [9, 38, 39, 41].

Our results demonstrate that the use of a standard controlled environment exposure for sample collection improves the reliability of measurement of molecules that could afterwards been used as potential pain biomarkers. These potential biomarkers could help stratify patients into subgroups based on the severity of pain or treatment response. This could enable more personalized medical care tailored to each patient’s needs.

### Limitations

Small sample size is the main limitation of the present study, although this sample offers sufficient statistical power. Also, as in our previous study [11], anxiety, stress, and sleep quality of subjects in this study had not been assessed directly, being possible that these variables may have influenced the biomarkers concentrations. To mitigate their influence, we narrowed the age range, excluded individuals with a history of significant medical conditions, psychiatric disorders, or ongoing psychotherapy, and allowed ample time before data collection to reduce the potential impact of the awakening response [11].

Another limitation is that three of the subjects included in the study were under systemic treatment (one with levothyroxine, another with mesalazine and another with ferrous sulphate supplementation), nevertheless, after reviewing the relevant literature on these drugs, we considered these drugs were unlikely to influence the results of biomarkers in saliva. Therefore, the saliva samples from these volunteers were not excluded from the study.

## Conclusion

In summary, we found that the reproducibility of the measurement of sTNFαRII, testosterone, and sAA in saliva in healthy subjects improved when analyzed after collection after exposure under controlled standard environmental conditions for 30 min. Cortisol reproducibility did not change. Also, the intersession variability improved in all biomarkers under CAC. Based on the results of this study and taking into consideration the findings of previous research conducted by our group, it can be suggested that sTNFαRII is the most robust biomarker. Environmental factors such as temperature and humidity affect the reproducibility of the different ocular pain biomarkers. The outcomes of this study will help clarify how the implementation of controlled environmental conditions enhances the utility of these molecules as biomarkers for chronic ocular pain. Thus, we recommend using controlled environmental conditions when planning clinical studies on saliva pain biomarkers, at least for the ones addressed in this study.

In accordance with the results obtained in this study, the use of standard controlled environments improves the reliability of salivary molecules which could be used as potential ocular pain biomarkers.

## Supporting information

S1 Table Saliva sample results in healthy subjects in the controlled environmental research laboratory. The data has been uploaded to the following repository: Figshare_My data; 2023. doi:10.6084/m9.figshare.24299155.
(XLSX)
